# Enhancing capacity of Zimbabwe’s health system to respond to climate change induced drought: a rapid nutritional assessment

**DOI:** 10.11604/pamj.2021.40.113.30545

**Published:** 2021-10-21

**Authors:** Zvanaka Sithole, Tasiana Nyadzayo, Trevor Kanyowa, Joyeux Mathieu, Tinashe Kambarami, Mildred Nemaramba, Ruth Machaka, Hana Bekele, Handrea Njovo, Alex Gasasira

**Affiliations:** 1 World Health Organization, Harare, Zimbabwe,; 2 Ministry of Health and Child Care, Harare, Zimbambwe,; 3 United Nations Children's Fund, Harare, Zimbabwe

**Keywords:** Health system’s preparedness, climate change, descriptive cross-sectional, Zimbabwe

## Abstract

**Introduction:**

Zimbabwe experienced the negative effects of the devastating cyclone Idai which affected several districts in the country, and the drought due to low rainfall that has affected the whole country. As a result of these catastrophes, the food and nutrition security situation in the country has deteriorated. For this reason, we carried out a rapid assessment of the health facilities in 19 sampled high global acute malnutrition and high food insecurity districts from the ten provinces of Zimbabwe to ascertain the preparedness of the facilities to respond to drought effects.

**Methods:**

we conducted a rapid nutritional assessment in 19 purposely selected districts with highest rates of global acute malnutrition from the 10 provinces of Zimbabwe. From these districts, we selected a district hospital and a rural health facility with high number of acute malnutrition cases. We adapted and administered the WHO recommended checklist (Multi-Cluster/Sector Initial Rapid Assessment (MIRA) as the assessment tool. We used STATA to generate frequencies, and proportions.

**Results:**

about 94% (16/19) of the districts had less than 50% health workers trained to manage acute malnutrition. A total of 26% (5/19) of the district hospitals and 32% (6/19) of the primary health care facilities were not admitting according to integrated management of acute malnutrition (IMAM) protocol. Twelve districts (63%) had none of their staff trained in infant and young child feeding (IYCF), 58% (11/19) had no staff trained in growth monitoring and 63% (12/19) of the districts had no trained staff in baby friendly hospital initiative (BFHI). A total of 60% of the provinces did not have combined mineral vitamin mix stocks, 80% had no resomal stocks, 20% did not have micronutrient powder stocks and 30% had no ready to use supplementary food stocks in all their assessed facilities. Fifty percent (50%) of the health facilities were not adequately stocked with growth monitoring cards. Manicaland had the least (20%) number of health facility with a registration system to notify cases of malnutrition.

**Conclusion:**

we concluded that the Zimbabwe health delivery system is not adequately prepared to respond to the effects of the current drought as most health workers had inadequate capacity to manage acute malnutrition, the nutrition surveillance was weak and inadequate stocks of commodities and anthropometric equipment was noted. Following this, health workers from six of ten provinces were trained on management of acute malnutrition, procurement of some life -saving therapeutic and supplementary foods was done. We further recommend food fortification as a long-term plan, active screening for early identification of malnutrition cases and continuous training of health workers.

## Introduction

Zimbabwe is experiencing the negative effects of the devastating cyclone Idai which affected several districts in the country, and the drought due to low rainfall that has affected the whole country. Poor rains experienced in Zimbabwe between 2018 and May 2019 [1], exacerbated by the Cyclone Idai of March 2019 affected food harvests for much of the population of the country. As a result of these catastrophes, the food and nutrition security situation in the country has been deteriorating. Zimbabwe has a rainfed Agro-based economy [2], where majority of the population derives its livelihood and food security from agriculture-based activities. The drought currently being experienced in the country has caused large-scale crop failure and reduced livestock production in many parts of the country. It was predicted that nearly six million rural populations would be food insecure at the peak hunger season between October 2019 and March 2020 and would need food assistance [3]. Resultant to the current food security and economic environment, the prevalence of global acute malnutrition (GAM) in children below the age of five years has increased to 3.6 percent from 2.5 percent reported in 2018 with eight districts having a prevalence of above 5% [3].

The prevalence of acute malnutrition is likely to increase with worsening food insecurity, compounded by the environment of high food prices, making access to basic food commodities difficult by most of the population in Zimbabwe [4,5]. The drought also has a bearing on the nutrition and health of the population, and in turn having a major impact on all determinants of health and health care services especially where the health sector is already overwhelmed in the delivery of its service [6,7]. For a nation to be healthy, it is important to eradicate hunger and all forms of malnutrition. As such, in drought and disaster situations, the burden of the problem should be established and effective coordination and adequate resourcing of nutrition interventions in the country becomes paramount [8,9]. Responding to the effects of drought in Zimbabwe improves the maternal, infant and young child nutrition. It is against this background that we undertook a comprehensive nutrition assessment to determine the Zimbabwe Health Sector Preparedness to respond to drought effects and manage severe acute malnutrition. Specifically, we assessed the capacity of health workers to manage acute malnutrition and provide infant and young child feeding (IYCF) package. Using the World Health Organization (WHO) checklist (multi-cluster/sector initial rapid assessment (MIRA), we also assessed the commodity status for management of acute malnutrition and assessed the nutrition surveillance. Actions from this assessment findings partly contribute towards the achievement of the WHO global targets of 2025 of addressing the double burden of malnutrition [10,11].

## Methods

**Study design:** we conducted a rapid nutritional assessment among health care workers in 19 districts with the highest rates of global acute malnutrition to determine the country´s preparedness to respond to effects of the current drought.

**Study setting:** the study was conducted in all 10 provinces of the country. A total of 19 districts with the highest rates of global acute malnutrition(GAM) were assessed.

**Source population:** health workers from the selected districts were the source population.

**Sample size calculation:** we determined the sample size by using the single population proportion formula assuming the proportion of health professionals who needed training on disaster preparedness and response as 50% [12], at 5% level of significance. The non-response rate of 5% was factored. The minimum sample size of 108 was calculated.

**Sampling procedure:** we purposively selected two districts from each of the ten provinces of Zimbabwe. We selected districts that had the highest global acute malnutrition (GAM) prevalence (>5%). From each district, we selected a district hospital and one rural health facility with high acute malnutrition cases according to the District Health Information System (DHIS 2). Purposive sampling was used to come up with valuable information and reducing the chances of potential bias. We interviewed health worker who were on duty on the date of assessment.

**Data collection:** we adapted and administered the WHO recommended checklist (multi-cluster/sector initial rapid assessment (MIRA) as the assessment tool. We conducted a two-day training on how to use the data collection tools. Data were collected using handheld android devices installed with a KOBO collect application and was immediately uploaded to a central server. We collected data from health facility personnel from the selected sites using key informant interviews. Direct observations of the status of infrastructure, equipment and drugs were done to triangulate data collected from the key informants. The exercise was conducted by five data collection teams made up of officers from the Ministry of Health and Child Care, Harare and Bulawayo City Health departments and World Health Organization. Key informant interviews were conducted at the family and child health (FCH) department, pediatric ward, pharmacy departments and antenatal care (ANC) units. The data collection exercise was done over a period of five days.

**Data analysis:** we captured data from the assessment, cleaned and analyzed it using STATA (version 13). We used descriptive statistics using frequencies, means and cross tabulations to draw summary measures. Missing data were excluded from the analysis.

## Results

We successfully assessed 19 districts from the 10 provinces of Zimbabwe. A total of 38 health care facilities (19 district hospitals and 19 primary health care facilities) from these districts were assessed on preparedness to respond to drought effects. Nineteen districts out of the sampled 20 were assessed. The assessment recruited 108 participants, and of these majority (60%) were nurses.

**Capacity of health care workers to manage acute malnutrition:** health care workers were assessed on capacity to manage acute malnutrition. Among the assessed districts, no health worker had received training on management of acute malnutrition in Lupane and Chirumhanzu. Almost all districts (94%) reported having trained staff, and all these districts reported having less than 50% of their health workers trained on managing acute malnutrition (Table 1).

**Table 1:** health workers trained on management of acute malnutrition by District, November 2019

District	Staff expected to be trained (N)	Staff trained (n)	Proportion trained (%)
Beitbridge	125	45	36
Bindura	234	36	15
Binga	92	9	10
Bulawayo	146	7	5
Chirumhanzu	92	0	0
Chitungwiza	619	29	5
Gwanda	209	5	2
Harare	1291	64	5
Kariba	80	54	68
Lupane	105	0	0
Makoni	201	2	1
Masvingo	100	3	3
Mberengwa	91	2	2
Mudzi	78	3	4
Murehwa	111	30	27
Mutare	631	11	2
Rushinga	105	44	42
Sanyati	204	56	27
Zaka	130	7	5

**Health facilities admitting clients into integrated management of acute malnutrition (IMAM) programme according to protocol:** capacity of health care workers to manage acute malnutrition was also assessed by checking the proportion of health facilities admitting clients into the IMAM programme according to protocol. Generally, most of the assessed facilities were admitting according to the IMAM protocol with some exceptions (26%) at district level and (32%) at rural health facilities not admitting patients into programs according to protocol.

**Capacity of health care workers to provide infant and young child feeding (IYCF) services:** capacity of health care workers to provide IYCF services was assessed through checking the proportion of health care workers trained in Infant and Young Child Feeding in the last two years. A total of 12 districts (63%) had none of their staff trained in infant and young child feeding (IYCF). The other seven districts (37%) had less than 50% of their HCW trained. Beitbridge had the highest number of trained staff (38%) among others.

**Health care workers trained in growth monitoring by District:** majority (58%) of the districts had no staff trained in growth monitoring. For the districts with trained staff, the proportions were less than 30% (Table 2).

**Table 2:** proportion of health care workers trained in growth monitoring by district, November 2019

District	Staff expected to be trained (N)	Staff trained (n)	Proportion trained (%)
Beitbridge	125	30	24
Bindura	234	0	0
Binga	92	0	0
Bulawayo	146	5	3
Chirumhanzu	92	0	0
Chitungwiza	619	9	1
Gwanda	209	2	1
Harare	1291	4	0
Kariba	80	6	8
Lupane	105	0	0
Makoni	201	1	0
Masvingo	100	0	0
Mberengwa	91	0	0
Mudzi	78	13	17
Murehwa	111	30	27
Mutare	631	2	0
Rushinga	105	0	0
Sanyati	204	5	2
Zaka	130	0	0

**Proportion of staff trained in baby friendly hospital initiative (BFHI) in the last two years:** majority (63%) of the districts had no trained staff in BFHI. Beitbridge, Kariba and Masvingo district hospital had more than 80% of their staff trained (Table 3).

**Table 3:** proportion of staff trained in baby friendly hospital initiative (BFHI) in the last two years

District	Expected to be trained (n)	Staff trained (n)	Proportion trained (%)
Beitbridge	123	105	85
Bindura	308	0	0
Binga	133	25	19
Bulawayo	140	6	4
Chirumhanzu	79	0	0
Chitungwiza	592	0	0
Gwanda	210	144	69
Harare	1285	0	0
Kariba	69	56	81
Lupane	103	0	0
Makoni	202	26	13
Masvingo	114	96	84
Mberengwa	100	0	0
Mudzi	75	0	0
Murehwa	109	0	0
Mutare	442	0	0
Rushinga	148	0	0
Sanyati	203	0	0
Zaka	147	0	0

**Figure 1:**
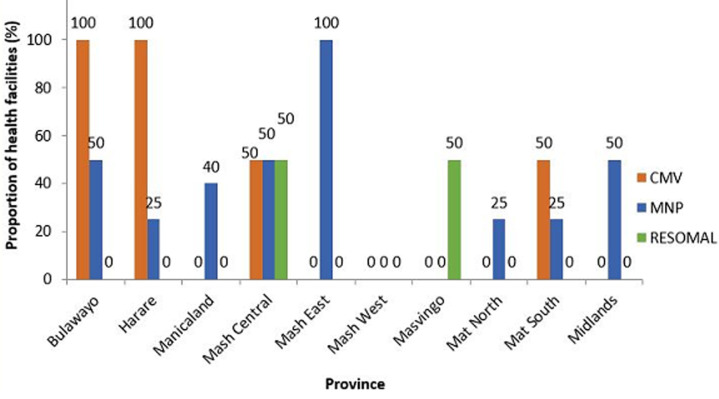
proportion of health facilities with combined mineral vitamin mix (CMV), micronutrient powder (MNP) and resomal per province, November 2019

**Figure 2:**
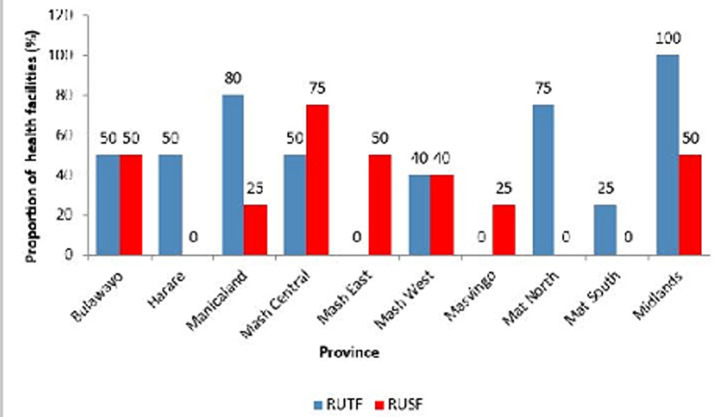
availability of RUTF and RUSF in the provinces, November 2019

**Commodity status for management of acute malnutrition:** a total of 60% of the provinces did not have combined mineral vitamin (CMV) mix stocks. Eighty percent (80%) of the provinces had no resomal stocks at all. Twenty percent (20%) of the provinces did not have MNP stocks. Mashonaland West province had none of these three commodities in stock (Figure 1). Majority of the health facilities within the 10 provinces had ready to use therapeutic food (RUTF) stock outs with Mashonaland East and Masvingo having no RUTF stocks at all within their health facilities. Only Midlands had RUTF stocks in all its facilities. Thirty percent (30%) of the provinces had no RUSF stocks. Manicaland had stocks of RUTF in 80% of its facilities (Figure 2). Mashonaland Central and Masvingo province had stock outs of F100 in all health facilities visited. Mashonaland central also had F75 stock outs in all its assessed facilities.

**Availability of anthropometric equipment:** health facilities were assessed for availability of anthropometric equipment. The most commonly used type of scales were the salter scale and the mother child electronic scale. The beam and spring hanging scale and the tuber spring hanging scale were the least used. Only 17% (3/19) of the districts had balance scales.

**Height-boards**: the standardized length/height boards were the commonly used equipment for measuring length and height across all selected districts. All health facilities had at least one standard height-board.

**Mid upper arm circumference (MUAC) tapes:** majority of the assessed districts had adequate MUAC tapes though some facilities in Gwanda, Makoni, Lupane, Mberengwa, Murehwa and Sanyati had less than five MUAC tapes, most of which were worn out for routine clinical use.

**Salter and infant scales:** the most used type of scale at selected health facilities was the salter scale. The mother-to-child scale was not available in eight districts, while the remaining 11 districts had at least one scale per facility.

**Growth monitoring cards:** fifty percent of the health facilities were not adequately stocked with growth monitoring cards.

**Proportion of village health workers (VHW) trained in active screening and early management of acute malnutrition:** Mutare had the least number of health facilities (33%) with trained village health workers in active screening and early management of acute malnutrition. Of these districts very few village health workers who were submitting the active screening reports for malnutrition with Bindura, Chitungwiza and Rushinga not at all submitting their reports. The cited reasons for not submitting these reports were long distances, no transport, lack of knowledge and work overload.

**Proportion of health facilities with a registration system to notify cases of malnutrition, November 2019:** Manicaland had the least (20%) number of health facility with a registration system to notify cases of malnutrition. Sixty percent of the provinces had a system to notify cases of malnutrition in all their facilities.

**Health facilities taking, recording and a Anthropometric measurements:** about 85% of the 38 health facilities (19 districts and 19 primary health care facilities) were taking anthropometric measurements. Of these facilities 65% were recording measurements on the tally sheets. Anthropometric indicators were being analyzed by 63% of the facilities recording on tally sheets.

**Acute malnutrition case fatality rate by Province:** overall, the national case fatality rate (CFR) for Acute malnutrition was 1.2%. Matabeleland South recorded the highest case fatality rate (5%) which higher than the national average, whilst Mashonaland East had a 0% case fatality rate.

**Funding:** the study was funded by WHO through CERF funding.

**Availability of data and materials:** the data that support the findings of this study are available from the Ministry of Health and Child Care Zimbabwe, but restrictions applies to the availability of these data. Data are however available from the authors upon reasonable request and with permission from Ministry of Health Child Care Zimbabwe.

**Ethics approval and consent to participate:** we sought permission to conduct the rapid assessment from the ministry of health and childcare ethical review board. We also sought written informed consent from the health workers. Health workers had the choice to participate or with draw from the assessment at any given point. We always ensured strict confidentiality when handling data during all processes of data collection, capturing, analysis and storage. Questionnaires were kept under lock and key throughout the assessment. Findings from this assessment were only shared with relevant authorities.

## Discussion

As health needs increase during droughts, increased capacity among health workers on treatment, care and management of vulnerable populations (children and women) become a necessity [12]. Findings from the assessment show that the Zimbabwe health sector is not well-prepared to manage acute malnutrition, as most of its health care workers lack the capacity to manage acute malnutrition. The capacity ranges from increased needs for medical supplies and trained human resources to an effective nutrition surveillance to detect increases in malnutrition. Matabeleland South province recorded 5% malnutrition case fatality rate, higher than the national average. This could be due to inadequate care and treatment or other different reasons. These assessment findings are consistent with Berhanu *et al*. (2016) who also found out that health professionals in Ethiopia were inadequately trained to respond to disaster effects [12]. Promotion of IYCF practices within the community is critical yet it is worrisome to note that majority of the assessed facilities had a few of their health care workers who were adequately prepared or trained to build the capacity of community workers to address malnutrition. Findings from a study by Yusa *et al*. (2015) on effects of climate change induced drought showed that Infant and young child feeding in emergency (IYCF-e) support to parents and caregivers of children below the age of two years is compromised due to incapacitation of health care workers [13].

This assesses showed incapacitation of health workers to support IYCF-e. Regarding supplies and logistics, an increase in health needs has an impact on the case load of health facilities. Shortage of commodities like RUTF in this assessment may negatively affect the integrated management of acute malnutrition outcomes leading to non-compliance, relapse and longer length of stay in hospital. Of concern is Manicaland province that had 80% of the facilities short of RUFT considering that this is the province with the two districts affected by cyclone Idai. This challenge could be exacerbated by the prevailing droughts and requires comprehensive measures to address it. In other facilities the stock outs were due to the push supply chain strategy where decisions about when and how many quantities of products to be delivered are determined by anticipated customer demand. This system sometimes underestimates or overestimates the quantities of stocks required by health facilities, resulting in unnecessary stocks outs while other facilities are over stocked as there is no equitable distribution of commodities. Strong surveillance system help identify existing and emerging malnutrition cases [14,15]. The assessment showed that surveillance systems were weak, as observed by the small proportion of health workers submitting active screening reports. A few of the assessed facilities were analyzing anthropometric indicators. This compromises early case identification. The study assessed only health workers who were on duty, and this could have introduced some bias. However, only health workers from relevant departments were assessed.

## Conclusion

Following the findings from the rapid assessment conducted in 19 districts from the 10 provinces of Zimbabwe, we concluded that the Zimbabwe health delivery system is not adequately prepared to respond to the effects of the current drought due to various factors that include shortage of human resources with capacity to manage acute malnutrition and shortage of commodities among others. It was noted that most health workers have not been capacitated in the management of acute malnutrition among other programmes essential in addressing public health nutrition like IYCF. Knowledge gaps identified among health care workers on management of acute malnutrition cases and infant and young child feeding (IYCF) clearly shows how inadequately prepared the country is. We also concluded that there are no enough commodities to use in response to the effects of drought as commodities such as RESOMAL, F75, ready to use supplementary feed (RUSF), ready to use therapeutic feed (RUTF) and micronutrient powders were either inadequate or out of stock in most of the facilities visited. Infant and Young Child Feeding (IYCF) could be compromised as very few health care workers were trained in (IYCF) and majority of the health facilities did not have standard tools for growth monitoring, management of acute malnutrition and Infant and Young Child Feeding (IYCF) individual counselling registers. The assessed districts were representative as there were withdrawn from all the 10 provinces of the country and the findings could be generalized. Public health actions taken so far include training of health workers on management of acute malnutrition. A total of six of 10 provinces has received training om management of acute malnutrition. Again, procurement of some life -saving therapeutic and supplementary foods was done.

**Recommendations:** based on the assessment findings there is need to increase access to and efficient stock management of life-saving therapeutic and supplementary foods at health facilities and community levels. The Push supply chain strategy where decisions about when and how many quantities of products to be delivered are determined by the actual customer demand should be adopted to avoid stock outs. Case management for acute malnutrition should be strengthened, therefore on-job training of health staff on management of acute malnutrition, Infant and Young Child Feeding (IYCF) and IDSR should be conducted. Provision of community level infant and young child feeding in emergencies support to parents and care givers is needed. There is need to strengthen food fortification for increased consumption of fortified staple foods. Active screening for early identification and referral of children with acute malnutrition through community-based health workers should be implemented. There is need to strengthen nutrition surveillance/ early warning through training of relevant health staff in all provinces and local authorities in IDSR. There is need to strengthen documentation of nutrition activities carried out at facility level, data collection, reporting and analysis of data to improve evidence-based decision making and effective nutrition response. The Ministry of Health and Child Care (MoHCC) with support from partners need to consider restocking of standard anthropometric equipment and growth monitoring cards in all health facilities. There is urgent need to provide some health facilities with infant scales. In addition, scales in health facilities need maintenance to ensure collection of accurate data for nutrition surveillance and growth monitoring activities.

### What is known about this topic

- It is already known that the country is experiencing some effects of climate change in addition to other natural disasters and the country must respond to these effects.

### What this study adds

- As such, in drought and disaster situations, the preparedness of the country has to be assessed; the rapid assessment provides information on the status of preparedness of the Zimbabwe's health system to respond to climate induced drought; it also enhances the capacity of provincial and district teams to respond to emergencies and provides evidence bases information for mobilization of resources for nutrition interventions.
